# Time trends in survival and causes of death in multiple myeloma: a population-based study from Germany

**DOI:** 10.1186/s12885-023-10787-5

**Published:** 2023-04-06

**Authors:** Christine Eisfeld, Hiltraud Kajüter, Lennart Möller, Ina Wellmann, Evgenii Shumilov, Andreas Stang

**Affiliations:** 1 Cancer Registry of North Rhine-Westphalia, Bochum, Germany; 2 Department of Medicine A, Hematology, Oncology and Pneumology, University Hospital Münster, Münster, Germany; 3 Institute of Medical Informatics, Biometry and Epidemiology, University Hospital Essen, Essen, Germany; 4 School of Public Health, Department of Epidemiology, Boston University, Boston, USA

**Keywords:** Multiple Myeloma, Survival, Relative Survival, Conditional Survival, Registries, Causes of death, Germany

## Abstract

**Background:**

Steady evolution of therapies has improved prognosis of patients with multiple myeloma (MM) over the past two decades. Yet, knowledge about survival trends and causes of death in MM might play a crucial role in long-term management of this patient collective. Here, we investigate time trends in myeloma-specific survival at the population level over two decades and analyse causes of death in times of prolonged survival.

**Methods:**

Age-standardised and age group-specific relative survival (RS) of MM patients aged < 80 years at diagnosis was estimated for consecutive time periods from 2000–2019 using data from the Cancer Registry of North Rhine-Westphalia in Germany. Conditional RS was estimated for patients who already survived one to five years post diagnosis. Causes of death in MM patients were analysed and compared to the general population using standardised mortality ratios (SMR).

**Results:**

Three thousand three hundred thirty-six MM cases were included in the time trend analysis. Over two decades, age-standardised 5-year RS increased from 37 to 62%. Age-specific survival improved from 41% in period 2000–2004 to 69% in period 2015–2019 in the age group 15–69 years, and from 23 to 47% in the age group 70–79 years. Conditional 5-year RS of patients who survived five years after diagnosis slightly improved as compared to unconditional 5-year RS at diagnosis. MM patients are two times more likely to die from non-myeloma malignancies (SMR = 1.97, 95% CI 1.81–2.15) and from cardiovascular diseases (SMR = 2.01, 95% CI 1.86–2.18) than the general population.

**Conclusions:**

Prognosis of patients with MM has markedly improved since the year 2000 due to therapeutic advances. Nevertheless, late mortality remains a major concern. As survival improves, second primary malignancies and cardiovascular events deserve increased attention.

**Supplementary Information:**

The online version contains supplementary material available at 10.1186/s12885-023-10787-5.

## Background

Multiple myeloma (MM) relates to the group of malignant plasma cell neoplasms and represents one of the most common haematological malignancies. The annual crude incidence rate of MM is about 8/100,000, increasing with age and accounting for 1.3% of all cancer diagnoses and for 1.8% of cancer related deaths in Germany [1]. Since 1990, the MM incidence rate increased in countries reporting cancer incidence data, a trend attributable to factors such as population growth, change in age structure, diagnostic improvements, and others [2, 3]. Although MM remains incurable in most cases, clinical studies have shown improvement of survival over the past decades [4]. Accordingly, evidence coming from well-established cancer registries has shown an increase of survival estimates on the population level since the 1990s [5–10]. Along this line, time to relapse or progression has been prolonged, due to pharmacological advances in conjunction with continuous therapy. Introduction of immune-based therapies such as anti-CD38 antibodies, next generations of proteasome inhibitors and immunomodulating agents has resulted in higher remission rates even in later lines of therapy [11]. For Germany, population-based MM survival data are available for limited time periods only. The national health authority “Robert Koch-Institut” reported improvement of 5-year relative survival (RS) from 46% in men and women in 2007–2008 to 54% in men and 56% in women in 2017–2018 [12]. To our knowledge, there are so far no population-based studies on MM survival over more than one decade in Germany. Nevertheless, as the patient collective included in randomised clinical trials (RCTs) does not fully represent patients treated in routine practice, the question of whether survival benefits are evident outside of clinical trials is of high practical relevance [13].

As survival improves, it is important that physicians become more aware of comorbidities and non-myeloma mortality risks emerging within the course of MM. Firstly, an increase of mortality from age-related diseases is to be expected in an aging population. Secondly, after prolonged exposition to cytostatic and/or immunomodulatory therapy, the risk of secondary primary malignancies (SPM) as well as the risk of fatal outcomes due to late-occurring side effects and cumulative toxicity might increase [14–16]. Hence, a comprehensive analysis of the causes of death among MM patients provides valuable information about potentially fatal secondary risks outside of RCTs and might contribute to their early prevention.

Here, we report incidence rates and provide up-to-date, age- and sex-specific estimates of RS for MM patients based on data from the largest population-based cancer registry in Germany. In the light of a changing treatment landscape, we analysed time trends in survival from 2000 to 2019 in a subset of the registry. In addition, we computed conditional RS estimates, providing information for patients and physicians about the current prognosis during follow-up. Furthermore, the distribution of causes of death in MM patients was analysed and compared with that in the general population*.*

## Material and methods

### Cancer registration and study population

North Rhine-Westphalia (NRW) is the most populated federal state in Germany (18 million inhabitants). Cancer reporting to the Cancer Registry of NRW is mandatory since 2005. From 1993 to 2004, the cancer registry covered only a subset of the NRW population, the administrative district of Münster (MS), with a population of 2.6 million people. Since 2008, data are available for whole NRW in sufficient quality. We included newly diagnosed cases with MM based on the International Classification of Disease 2010 (ICD-10) code C90. Patients living in MS and diagnosed between 1995–2019 were included in the MS cohort. Patients living in NRW and diagnosed between 2010–2019 were included in the NRW cohort. Comprehensive mortality follow-up for cancer patients was routinely assessed through validated record linkage with electronic reports on all deceased individuals in NRW obtained from the population registry [17].

### Statistical methods

Crude and age-standardised incidence rates were calculated for NRW and for MS with all cases diagnosed in the respective period as nominator and the sum of the annual mid-year population as denominator. Age-standardisation was performed using the old European Standard [18].

To estimate cancer-specific survival we calculated 5-year RS. RS for a calendar period is defined as the ratio of the observed survival time of MM patients (absolute survival) and the expected survival time of the general population of the same age, sex, and calendar period [19]. This can be interpreted as the expected survival of patients with cancer under the hypothetical assumption that cancer is the only cause of death [20]. Survival time per patient was the time interval between the date of diagnosis and death or end of the follow-up in 2019. Expected survival was estimated by the Ederer II method based on life tables of NRW and MS [20, 21]. We excluded subjects from the survival analysis if their diagnosis was notified by death certificate only (DCO). Survival analysis was restricted to patients aged 15–79 years at diagnosis, as the proportion of DCO increased with age, leading to overestimation of survival in older age groups.

RS was calculated using the period approach, since it provides more up-to date survival estimates than the traditional cohort approach and therefore enables detection of changes in survival timely [22]. We illustrated the principle of data use in Supplementary Figure S1 (Additional file 1). For example, for estimating five-year survival for calendar period 2015–2019 in the MS cohort, we used survival data of patients diagnosed 2010–2019. More precisely, in addition to those diagnosed in the period 2015–2019, the patients who survived until 2015 contributed (left truncated) their survival experience to the analysis as well. We estimated crude RS and age-specific RS for the age groups 15–69 years and 70–79 years. To allow for comparison across time periods and cohorts, RS was age-standardised according to the International Cancer Survival Standard (ICSS) 1, using four age categories (15–49 years, 50–59 years, 60–69 years, 70–79 years) [23].

For the analysis of the time trend of survival, five-year RS was estimated for periods 2000–2004, 2005–2009, 2010–2014, and 2015–2019 in the MS cohort. In the NRW cohort, five-year RS was estimated for the period 2015–2019. Additionally, in the NRW cohort we estimated five-year conditional RS after 1 to 5 years post diagnosis. More specifically, conditional RS is the RS given the patient already has survived for a specific time after diagnosis. For this purpose, the analysis was restricted to patients who survived at least one, two, three, four, or five years, respectively, and survival time per patient was calculated by the difference between the respective time point and death or right censoring whatever came first. The calendar period of interest for conditional RS estimation was 2015–2019.

Causes of death were analysed in the NRW cohort without age restrictions. Causes of death were arranged by ICD-10 chapter and mortality from selected causes of death was compared with that in the general population. For this purpose, standardised mortality ratios (SMR) and corresponding confidence intervals were calculated as the ratio of observed deaths to expected deaths if the age-sex specific mortality rates were the same as those of the standard population of NRW [24]. Calculation of SMRs was performed excluding DCO cases.

All analyses were performed with „R “, version 3.6.2, using the package „periodR “ for survival analysis [25].

## Results

### Patient characteristics

During the years 2010 to 2019, 14,815 MM cases were registered in NRW. During the years 1995 to 2019, 4702 MM cases were detected in MS, with case numbers continuously increasing over time from 769 cases between 2000–2004 to 1166 cases between 2015–2019. In the same time span, age-standardised incidence rates slightly increased from 4.4/100,000 (95% CI 4.1–4.7) to 5.3/100,000 (95% CI 5.0–5.6). We present the age distributions and incidence rates in the unrestricted populations in Supplementary Table S1 (Additional file 1). After restricting the analysis to patients 15–79 years old, the proportions of DCO cases were 14% in the NRW cohort and 7% in the MS cohort. 9513 and 3336 cases remained for the survival analysis in the NRW and in the MS cohort, respectively. The age and sex distributions were similar in both cohorts (Table 1).

**Table 1:** Characteristics of registered newly diagnosed multiple myeloma cases for survival analysis

	North Rhine-Westphalia (NRW)		Administrative district of Münster (MS)	
	Diagnosis 2010–2019		Diagnosis 1995–2019	
	15–79 years		15–79 years	
	Men	Women	Men	Women
Person years at risk (million)	70.8	72.4	25.7	26.3
Cases				
Including DCO	6348	4706	2026	1561
Study population for survival analysis (excl. DCO)	5488	4025	1888	1448
Age at diagnosis, incl. DCO (%)				
Median (years)	68	69	68	69
15–69 years	3492 (55.0)	2373 (50.4)	1140 (56.3)	839 (53.7)
70–79 years	2856 (45.0)	2333 (49.6)	886 (43.7)	722 (46.3)
Age at diagnosis, excl. DCO (%)				
Median (years)	67	68	67	68
15–69	3181 (58.0)	2184 (54.3)	1092 (57.8)	802 (55.4)
70–79	2307 (42.0)	1841 (45.7)	796 (42.2)	646 (44.6)
Histologically verified, excl. DCO (%)	3903 (71)	2797 (69)	1329 (70)	1010 (70)
Only cytologically verified, excl. DCO (%)	418 (8)	294 (7)	129 (7)	135 (9)

The person years at risk in NRW and MS are the sum of the annual mid-year population for the years 2010–2019 and 1995-2019, respectively

*Abbreviations: DCO* death certificate only

### Survival outcomes

**Fig. 1:**
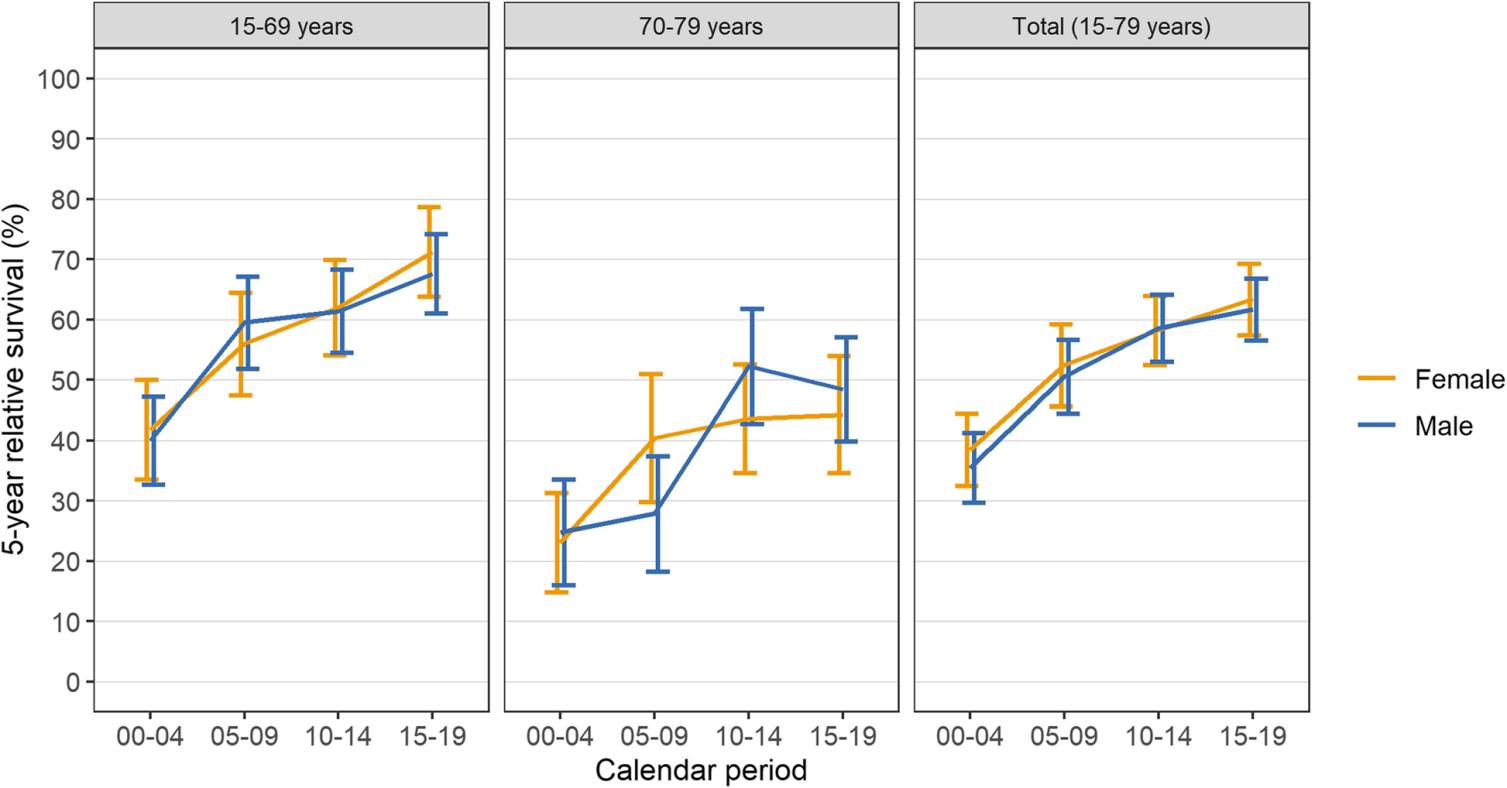
Age-specific and age-standardised 5-year relative survival for patients with multiple myeloma. Patients diagnosed 1995–2019 living in the administrative district of Münster were included. Error bars indicate 95% confidence intervals

We investigated temporal trends of survival using data from the MS cohort, allowing analysis over two decades. Five-year RS estimates are presented in Fig. 1 and in Table 2. Overall, age-standardised 5-year RS increased by 25.7 percentage points from 36.7% (95% CI 32.5–40.9) in period 2000–2004 to 62.4% (95% CI 58.5–66.3) in period 2015–2019. For men, age-standardised 5-year RS was 35.5% in 2000–2004 and then increased to 50.5% in 2005–2009, 58.6% in 2010–2014, and 61.7% in 2015–2019. For women, age-standardised 5-year RS was 38.5% in 2000–2004 and then increased to 52.4% in 2005–2009, 58.2% in 2010–2014 and 63.4% in 2015–2019. Stratified by two age groups, five-year RS of patients aged 15–69 years improved from 40.7% (95% CI 35.3–46.2) in period 2000–2004 to 69.2% (95% CI 64.2–74.1) in period 2015–2019, for patients aged 70–79 years five-year RS improved from 23.4% (95% CI 17.5–29.4) to 46.8% (95% CI 40.4–53.2) in the corresponding time periods. Minor survival differences between men and women in our analysis are not consistent and likely represent random variation.

**Table 2:** Five-year relative survival of patients with multiple myeloma

	5-year relative survival (95% CI)			
Calendar period	2000–2004	2005–2009	2010–2014	2015–2019
Men				
Study population (15–79 years)				
Crude	33.6 (28.1–39.2)	47.6 (41.4–53.9)	57.5 (51.9–63.1)	59.4 (54.0–64.7)
Age standardised	35.5 (29.8–41.3)	50.5 (44.5–56.6)	58.6 (53.0–64.1)	61.7 (56.5–66.8)
15–69 yrs	39.9 (32.7–47.2)	59.5 (51.8–67.2)	61.4 (54.5–68.3)	67.6 (61.0–74.2)
70–79 yrs	24.8 (16.0–33.6)	27.9 (18.3–37.5)	52.3 (42.8–61.8)	48.5 (39.9–57.1)
Women				
Study population				
Crude	33.5 (27.5–39.5)	49.5 (42.8–56.2)	53.4 (47.4–59.5)	59.9 (53.8–66.1)
Age standardised	38.5 (32.5–44.4)	52.4 (45.6–59.2)	58.2 (52.5–63.9)	63.4 (57.4–69.3)
15–69 yrs	41.8 (33.5–50.0)	56.0 (47.5–64.4)	62.0 (54.1–69.9)	71.2 (63.8–78.7)
70–79 yrs	23.1 (14.9–31.3)	40.4 (29.8–51.0)	43.7 (34.7–52.6)	44.3 (34.6–54.0)
Both				
Study population				
Crude	33.5 (29.4–37.6)	48.5 (43.9–53.1)	55.6 (51.5–59.7)	59.6 (55.5–63.7)
Age standardised	36.7 (32.5–40.9)	51.5 (46.9–56.1)	58.2 (54.2–62.3)	62.4 (58.5–66.3)
15–69 yrs	40.7 (35.3–46.2)	57.9 (52.3–63.6)	61.6 (56.4–66.8)	69.2 (64.2–74.1)
70–79 yrs	23.4 (17.5–29.4)	34.1 (26.9–41.3)	47.8 (41.3–54.4)	46.8 (40.4–53.2)

Patients diagnosed 1995–2019 living in the administrative district of Münster were included

*Abbreviations:* *CI* confidence interval

**Fig. 2:**
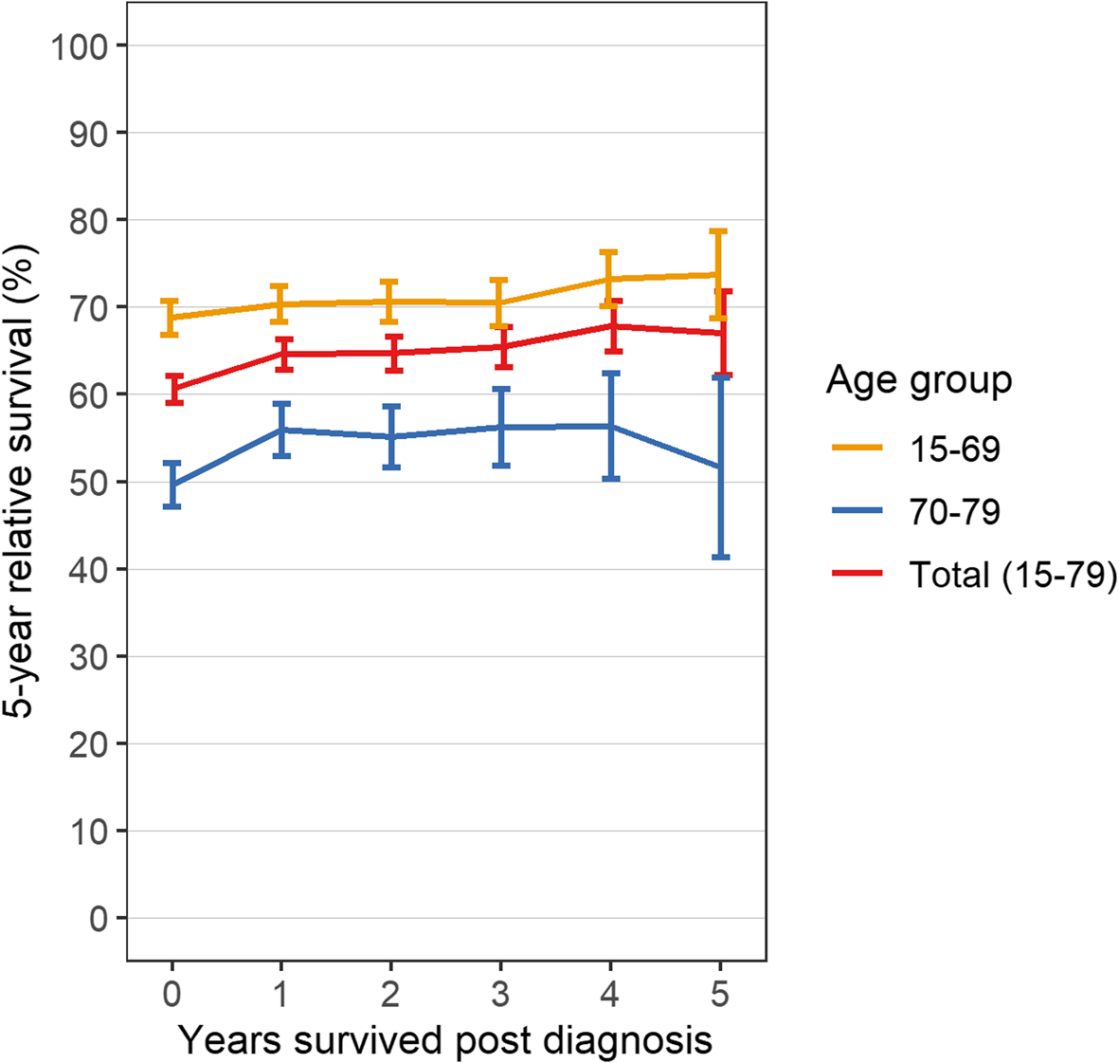
Five-year conditional relative survival of patients with multiple myeloma. Patients diagnosed 2010–2019 living in North Rhine-Westphalia were included. Error bars indicate 95% confidence intervals

Conditional RS was analysed for patients from the NRW cohort (Fig. 2, Table 3). As this cohort was considerably larger than the MS cohort, it enabled us to obtain more precise estimates for the recent period. Five-year RS for period 2015–2019 at diagnosis and conditional on surviving one, three, and five years post diagnosis was 60.6% (95% CI 59.1–62.2), 64.6% (95% CI 62.9–66.3), 65.5% (95% CI 63.1–67.8), and 67.0% (95% CI 62.3–71.8), respectively. The database used for deriving conditional RS estimates is shown in Supplementary Table S2 (Additional file 1).

**Table 3:** Five-year conditional relative survival at diagnosis of multiple myeloma and after surviving 1, 3, and 5 years

Age at diagnosis	Number of patients observed in period 2015–2019				Five-year conditional relative survival (95% CI)			
	At diagnosis	After surviving 1 year	After surviving 3 years	After surviving 5 years	At diagnosis	After surviving 1 year	After surviving 3 years	After surviving 5 years
Men								
Total (15–79 years)	4706	3777	2441	1305	60.9 (58.8–63.0)	64.9 (62.5–67.3)	65.5 (62.3–68.7)	68.3 (61.9–74.6)
15–69 yrs	2823	2344	1587	878	67.2 (64.6–69.7)	69.3 (66.5–72.0)	70.0 (66.5–73.5)	78.2 (72.4–83.9)
70–79 yrs	1883	1433	854	427	51.9 (48.4–55.4)	57.7 (53.5–61.9)	55.9 (49.4–62.3)	39.7 (23.3–56.2)
Women								
Total (15–79 years)	3492	2853	1863	1052	60.2 (57.9–62.5)	64.2 (61.7–66.8)	65.4 (62.0–68.8)	65.5 (58.3–72.8)
15–69 yrs	1972	1675	1111	679	71.1 (68.3–74.0)	71.9 (68.9–75.0)	71.3 (67.3–75.3)	67.4 (58.5–76.4)
70–79 yrs	1520	1178	752	373	47.3 (43.7–50.8)	54.0 (49.8–58.2)	56.4 (50.4–62.4)	61.5 (49.0–73.9)
Both								
Total (15–79 years)	8198	6630	4304	2357	60.6 (59.1–62.2)	64.6 (62.9–66.3)	65.5 (63.1–67.8)	67.0 (62.3–71.8)
15–69 yrs	4795	4019	2698	1557	68.8 (66.9–70.7)	70.4 (68.3–72.4)	70.5 (67.9–73.1)	73.7 (68.7–78.7)
70–79 yrs	3403	2611	1606	800	49.7 (47.2–52.2)	55.9 (53.0–58.9)	56.2 (51.8–60.6)	51.7 (41.4–62.0)

Patients diagnosed 2010–2019 living in North Rhine-Westphalia were included

*Abbreviations: CI* confidence interval

### Causes of death

There were 8654 deaths amongst 14,815 MM patients diagnosed between 2010–2019 in NRW. A selection of the most frequent causes of death among MM patients by ICD-10 chapter is depicted in Table 4. Besides 76.8% dying from MM, major causes of death in MM patients were cardiovascular diseases (7.0%) and non-myeloma malignancies (6.1%). MM patients are about two times more likely to die from cardiovascular diseases (SMR 2.01, 95% CI 1.86–2.18) and from non-myeloma malignancies (SMR 1.97, 95%-CI 1.81–2.15) than the general population. Additionally, mortality from diseases of the genitourinary system, infectious diseases, endocrine, nutritional and metabolic diseases and from gastrointestinal diseases was increased in MM patients as compared to the reference population (SMRs 3.97, 3.77, 3.04, and 1.72, respectively). SMR estimates stratified by sex are provided in Supplementary Table S3 (Additional file 1).

**Table 4:** Selected causes of death by ICD-10 chapter among patients with multiple myeloma

Cause of death (ICD-10 codes)	Observed No. of deaths (%)	Expected No. of deaths	SMR (95% CI)
Multiple myeloma and plasma cell neoplasms (C90)		-	-
incl. DCO	6650 (76.8)		
of which DCO	3119		
Cardiovascular diseases (I00-I99)	602 (7.0)	299	2.01 (1.86–2.18)
Non-myeloma malignancies (C00-D48, excl. C90)	528 (6.1)	268	1.97 (1.81–2.15)
Respiratory diseases (J00-J99)	161 (1.9)	80	2.01 (1.73–2.35)
Diseases of the genitourinary system (N00-N99)	95 (1.1)	24	3.97 (3.25–4.86)
Certain infectious and parasitic diseases (A00-B99)	90 (1.0)	24	3.77 (3.06–4.63)
Endocrine, nutritional and metabolic diseases (E00-E90)	71 (0.8)	23	3.04 (2.41–3.84)
Gastrointestinal diseases (K00-K93)	66 (0.8)	38	1.72 (1.35–2.18)
Mental and behavioural disorders (F00-F99)	30 (0.3)	32	0.94 (0.66–1.34)
Diseases of the nervous system and sensory organs (G00-H95)	25 (0.3)	29	0.86 (0.58–1.28)
Non-informative causes of death (R00-R94, R95-R99)	99 (1.1)	50	1.99 (1.64–2.43)
Unknown ^a^	130 (1.5)	-	-

Patients diagnosed 2010–2019 living in North Rhine-Westphalia were included

*Abbreviations: SMR* age-sex-standardised mortality ratio, *CI* confidence interval, *DCO* death certificate only

^a^ Confirmed deaths that could not be assigned to a cause of death

## Discussion

The results from this population-based study show that over the past twenty years, age-standardised 5-year relative survival of patients with MM under the age of eighty remarkably increased from 36.7% to 62.4%. While survival probabilities remained strongly dependent on age, we showed that survival improvement over time occurred in both age categories 15–69 years and 70–79 years.

Data from other population-based studies have shown that survival in MM has improved substantially over the last two decades. Turesson et al. have reviewed the available evidence coming from registry studies including data until the year of 2014 [6]. Pulte et al. examined trends in survival from 2002 to 2010 in a cohort from twelve regional cancer registries in Germany aged 15–74 years and reported an increase of 5-year RS from 47.3% to 53.8% [26]. Studies from the Netherlands and from New Zealand included patients diagnosed between 1989–2018 and 1990–2016, respectively, reporting that the main improvement of survival was achieved from 1999 onwards, that is, in the period covered by our analysis [9, 10]. Our 5-year RS estimates from the most recent period are overall comparable to data from other cancer registries, apart from differences in the age groups included [7, 8, 10].

The number and the efficacy of therapeutic substances for MM has increased dramatically. The introduction of the proteasome inhibitor bortezomib as well as immunomodulatory drugs thalidomide and lenalidomide and their European Medicines Agency approvals in the years 2004–2009 have changed treatment paradigms in MM. Since then, results from RCTs have shown that the use of novel agents, targeted therapies, and multidrug regimens in patients with MM has led to improvements of overall survival [27–31]. These improvements are consistent with the ongoing increase of RS since the years 2000–2004 shown in the data presented here.

Importantly, we observed improvement of survival estimates in the age group 70–79 years from 2000–2004 to 2010–2014, a subgroup which is often not well represented in clinical trials [32]. This is consistent with findings from other studies, reporting survival improvements in advanced age groups during the past 15–20 years [7, 10, 33]. From 2010–2014 to 2015–2019, we observed stagnation of survival estimates in this subgroup, although statistical precision is limited due to small numbers. A reason for this stagnation might be more rapid “real-world” dissemination of newly approved substances in younger patients as compared to elderly patients [32, 33]. Our findings should be verified in a larger dataset and in a more recent period.

Alternative or additional reasons for survival improvements may include novel diagnostic techniques and new diagnostic criteria released by the International Myeloma Working Group (IMWG) in 2014 that might have led to earlier diagnosis and thereby might have influenced survival time [34]. However, we did not observe major changes in the annual incidence rates following the publication of the new diagnostic criteria in our data. In the absence of population-based studies assessing the epidemiological impact of the IMWG update, we assume that the proportion of MM cases in whom the new diagnostic criteria has brought forward the time of diagnosis without therapeutic benefit (lead time bias) is small.

To our knowledge, this is the first study reporting estimates of conditional RS in MM in the era of effective multidrug therapies. We could show that, for patients from the NRW cohort, conditional 5-year RS slightly increased from 60.6% to 67.0% after five years already survived compared to diagnosis. Accordingly, previous population-based analyses have shown that conditional RS slightly increased over 5 years [35, 36], whereas results from a clinical study suggest that conditional overall survival remained stable after one, three, and five years survived [37]. Overall, the results are in line with registry-based studies investigating long-term survival, showing that the evolution of MM is not precluded after 5 years, but late mortality is an ongoing issue [8]. Through dynamic assessment of cause-specific survival we provide valuable information for patients and clinicians on how prognosis develops over the course of the disease.

As MM survivorship increases following the introduction of novel therapies, mortality from SPM and late side effects of treatment as well as from fatal age-related diseases becomes an issue. In our analysis, MM patients were more likely to die from cardiovascular diseases or from non-myeloma malignancies (including SPM) compared to the general population. While in earlier studies the overall risk of SPM was not increased in MM patients [38], population-based studies have shown an increase in the incidence of SPM in recent years due to longer survival and possibly linked to the administration of lenalidomide and melphalan [16, 39]. Consistent with our findings, an analysis of data from the Surveillance, Epidemiology, and End Results (SEER) Program found a proportion of non-myeloma cancer deaths in MM patients of 5.4% and showed a more than twofold increase in the risk of death from cardiovascular diseases in MM patients compared to the general population [15]. Our findings raise awareness of long-term risks and toxicities, highlighting the particular role of intensified monitoring and screening as well as prevention measures in this patient collective.

The strength of this study is the use of population-based data from one of Europe’s largest cancer registries, providing survival data of patients treated in Germany, where 90% of patients belong to one of the statutory health insurances, which in principle ensures equal access to cancer therapy. Using the relative survival approach, we were able to generate evidence about cancer-specific survival in addition to overall survival. In an aging patient population, comorbidities increasingly contribute to mortality and relying on overall survival only might underestimate benefits of therapy. Moreover, due to comprehensive mortality follow-up in the Cancer Registry of NRW, we were able to provide a detailed analysis of causes of death.

We recognise that our work has some limitations. First, for periods back into the past, we had to rely on data from a subset of the cancer registry only, and survival estimates were based on limited numbers of patients, especially when age stratification was applied. Second, as information about stage at diagnosis, prognostic factors, and therapy is incomplete in the registry dataset, evidence about the impact of new therapies on survival is merely indirect [40]. Third, a substantial proportion of incident cases were death certificate only (DCO) notified and had to be excluded from our survival analysis. One reason for the higher proportion of DCO cases in MM than in most solid tumors may be that some MM diagnoses, which rely on haematologists’ cytology reports, escape case notification by the registry, whereas case notification is largely complete when pathology reports are available. As DCO cases tend to be older and have a worse prognosis than cases notified at lifetime, exclusion of DCO cases might lead to overestimation of survival [41]. This is particularly important when comparing our results with population-based analyses from cancer registries, which report very small fractions of DCO cases. Furthermore, due to particularly high proportion of DCO in older age groups, we only included patients up to the age of 79 years. Consequently, our survival estimates cannot be applied to the age group 80 + , which is in fact a substantial proportion of patients in a disease with an average age at onset of 70–75 years [6]. Nonetheless, in our age-restricted survival analysis, the median age at diagnosis of 67–69 years is still relatively high as compared to clinical study populations [37]. As several new substances for MM treatment have been introduced in the last 5–10 years, information on survival in recent years is of particular interest to clinicians. Of note, our survival analysis covering the period 2015–2019 necessarily includes data from patients diagnosed and treated before 2015, thus, survival will likely be underestimated [8].

Regarding causes of death, there might be cases of misclassification. Renal failure (50 cases of death in our analysis) was formally assigned to ICD-10 chapter N, “Diseases of the genitourinary system”, but might instead be a consequence of active MM disease as the underlying cause of death. Death from amyloidosis (15 cases of death) was formally assigned to ICD-10 chapter E, “Endocrine, metabolic and nutritional diseases”. In the context of known underlying MM, most of these cases might have been instances of systemic light chain (AL) amyloidosis, for whom redistribution to MM as the underlying cause of death might have been correct.

## Conclusions

In conclusion, cancer-specific survival in MM has substantially improved over two decades, following the widespread use of new therapies. Additionally, diagnostic advances and changes in diagnostic criteria might have led to more patients being diagnosed in asymptomatic stages, contributing to the improvement of survival time shown here. Nevertheless, there is excess mortality compared to the general population throughout the course of the disease. With improving prognosis, clinicians should pay attention to second primary malignancies and cardiovascular risks.

## Supplementary Information

**Additional file 1:** **Supplementary Figure S1.** Schematic illustration of data use for estimation of five-year survival by period analysis for the period 2015-2019 (solid frame). The numbers within the cells indicate the follow-up years following diagnosis. Adapted from [20]. **Supplementary Table S1.** Age distribution and incidence rates per 100,000 person-years for multiple myeloma. **Supplementary Table S2.** Database used for estimation of conditional five-year survival (both sexes). The numbers within the cells indicate the summed up deaths/person years for each combination of follow-up year and calendar year of period. For example, in calendar year 2015, 133 deaths occured in 997 person years at risk during the first year of follow-up. **Supplementary Table S3.** Selected causes of death by ICD-10 chapter among patients with multiple myeloma, stratified by sex.

## Data Availability

The datasets generated and/or analysed during the current study are not publicly available for reasons of data protection, but are available from the corresponding author on reasonable request in accordance with relevant guidelines and regulations.

## Abbreviations

CI: Confidence interval
DCO: Death certificate only
ICD-10: International Classification of Disease 2010
ICSS: International Cancer Survival Standard
IMWG: International Myeloma Working Group
MM: Multiple myeloma
MS: Administrative district of Münster
NRW: North Rhine-Westphalia
RCTs: Randomised clinical trials
RS: Relative survival
SEER: Surveillance, Epidemiology, and End Results Program
SMR: Standardised mortality ratio
SPM: Second primary malignancies
