# Supplementary material for: Time trends in survival and causes of death in multiple myeloma: a population-based study from Germany
Source: BMC Cancer. 2023 Apr 6;23:317. doi: 10.1186/s12885-023-10787-5 (PMC10080943; doi:10.1186/s12885-023-10787-5)
Supplement: Supplementary file 1 — Additional file 1: Supplementary Figure S1. Schematic illustration of data use for estimation of five-year survival by period analysis for the period 2015-2019 (solid frame). The numbers within the cells indicate the follow-up years following diagnosis. Adapted from [20]. Supplementary Table S1. Age distribution and incidence rates per 100,000 person-years for multiple myeloma. Supplementary Table S2. Database used for estimation of conditional five-year survival (both sexes). The numbers within the cells indicate the summed up deaths/person years for each combination of follow-up year and calendar year of period. For example, in calendar year 2015, 133 deaths occured in 997 person years at risk during the first year of follow-up. Supplementary Table S3. Selected causes of death by ICD-10 chapter among patients with multiple myeloma, stratified by sex. [file 12885_2023_10787_MOESM1_ESM.pdf]

SUPPLEMENT

Supplementary Figure S1

Schematic illustration of data use for estimation of five-year survival by period analysis for the period 2015-2019 (solid frame). The numbers within the cells indicate the follow-up years following diagnosis. Adapted from [20].

Calendar year of diagnosis	Calendar year of follow-up: 2010	2011	2012	2013	2014	2015	2016	2017	2018	2019
2010	1	1-2	2-3	3-4	4-5	5				
2011		1	1-2	2-3	3-4	4-5	5			
2012			1	1-2	2-3	3-4	4-5	5		
2013				1	1-2	2-3	3-4	4-5	5	
2014					1	1-2	2-3	3-4	4-5	5
2015						1	1-2	2-3	3-4	4-5
2016							1	1-2	2-3	3-4
2017								1	1-2	2-3
2018									1	1-2
2019										1

Supplementary Table S1

Age distribution and incidence rates per 100,000 person-years for multiple myeloma

	North Rhine-Westphalia 2010-2019		Administrative district of Münster 1995-2019	
	Men	Women	Men	Women
Age distribution (%)				
<49	449 (6)	283 (4)	148 (6)	113 (5)
50-59	1149 (14)	765 (11)	335 (13)	210 (10)
60-69	1895 (23)	1327 (20)	658 (26)	516 (23)
70-79	2856 (35)	2333 (35)	886 (35)	722 (33)
80+	1736 (21)	2022 (30)	470 (19)	644 (29)
Total	8085	6730	2497	2205
DCO cases (% of incident cases)				
<49	19 (4)	6 (2)	3 (2)	2 (2)
50-59	86 (7)	49 (6)	12 (4)	5 (2)
60-69	207 (11)	134 (10)	33 (5)	30 (6)
70-79	549 (19)	492 (21)	90 (10)	76 (11)
80+	637 (37)	940 (46)	116 (25)	164 (25)
Total	1498 (19)	1621 (24)	254 (10)	277 (13)
Age-specific incidence rates (95% CI)				
<49	0.9 (0.8-0.9)	0.6 (0.5-0.6)	0.7 (0.6-0.8)	0.6 (0.5-0.7)
50-59	8.4 (7.9-8.9)	5.6 (5.2-6.0)	7.7 (6.8-8.5)	4.8 (4.2-5.5)
60-69	19.6 (18.7-20.5)	12.8 (12.1-13.4)	19.0 (17.6-20.5)	13.9 (12.7-15.1)
70-79	36.9 (35.5-38.2)	24.6 (23.6-25.6)	38.4 (35.9-41.0)	23.1 (21.4-24.8)
80+	48.1 (45.8-50.4)	30.4 (29.1-31.7)	51.5 (46.9-56.2)	31.5 (29.1-34.0)
Overall incidence rates (95% CI)				
crude	9.3 (9.1-9.5)	7.4 (7.2-7.6)	7.8 (7.5-8.2)	6.6 (6.3-6.9)
age-standardised	6.1 (6.0-6.2)	4.0 (3.9-4.1)	6.1 (5.8-6.3)	4.0 (3.8-4.1)

Abbreviations: DCO, death certificate only; CI, confidence interval

Supplementary Table S2

Database used for estimation of conditional five-year survival (both sexes). The numbers within the cells indicate the summed up deaths / person years for each combination of follow-up year and calendar year of period. For example, in calendar year 2015, 133 deaths occured in 997 person years at risk during the first year of follow-up.

			Calendar year of period				
			2015	2016	2017	2018	2019
Year of follow-up	1	At diagnosis	133/997	137/1016	143/1019.5	165/995.5	147/932
	2		90/795.5	68/846	103/896.5	81/863.5	88/837
	3		85/696.5	89/712	72/763.5	79/799.5	78/782.5
	4		65/600	84/623.5	70/618.5	74/689	59/720.5
	5		55/505.5	65/535	47/536.5	61/549.5	59/624
	1	1-year survivors	87/792.5	67/845	102/895.5	81/863.5	85/834
	2		85/696.5	89/712	72/763.5	79/799.5	78/782.5
	3		65/600	84/623.5	70/618.5	74/689	59/720.5
	4		55/505.5	65/535	47/536.5	61/549.5	59/624
	5		23/222	39/445.5	48/478.5	47/483.5	48/496.5
	1	3-year survivors	64/599	84/623.5	69/617.5	73/688	58/718.5
	2		55/505.5	65/535	47/536.5	61/549.5	59/624
	3		23/222	39/445.5	48/478.5	47/483.5	48/496.5
	4		0	26/203	31/404	43/432.5	54/438
	5		0	0	20/182.5	40/371	35/379.5
	1	5-year survivors	23/222	39/455.5	48/478.5	46/482.5	47/495.5
	2		0	26/203	31/404	43/432.5	54/438
	3		0	0	20/182.5	40/371	35/379.5
	4		0	0	0	17/163	22/327
	5		0	0	0	0	18/150.5

Supplementary Table S3

Selected causes of death by ICD-10 chapter among patients with multiple myeloma, stratified by sex

Cause of death (ICD-10 codes)	Men		Women	
	Observed No. of deaths (%)	SMR (95% CI)	Observed No. of deaths (%)	SMR (95% CI)
Multiple myeloma and plasma cell neoplasms (C90)				
incl. DCO	3421 (73.9)	-	3229 (80.2)	-
of which DCO	1498		1621	
Cardiovascular diseases (I00-I99)	358 (7.7)	2.00 (1.80-2.22)	244 (6.1)	2.04 (1.80-2.31)
Non-myeloma cancer (C00-D48, excl. C90)	338 (7.3)	1.93 (1.74-2.15)	190 (4.7)	2.04 (1.77-2.35)
Respiratory diseases (J00-J99)	102 (2.2)	1.94 (1.60-2.35)	59 (1.5)	2.16 (1.67-2.79)
Diseases of the genitourinary system (N00-N99)	47 (1.0)	3.37 (2.53-4.48)	48 (1.2)	4.81 (3.63-6.39)
Certain infectious and parasitic diseases (A00-B99)	53 (1.1)	3.71 (2.83-4.85)	37 (0.9)	3.86 (2.79-5.32)
Endocrine, nutritional and metabolic diseases (E00-E90)	39 (0.8)	2.88 (2.11-3.95)	32 (0.8)	3.26 (2.31-4.61)
Gastrointestinal diseases (K00-K93)	38 (0.8)	1.62 (1.18-2.22)	28 (0.7)	1.87 (1.29-2.70)
Mental and behavioural disorders (F00-F99)	15 (0.3)	0.90 (0.54-1.49)	15 (0.4)	0.98 (0.59-1.63)
Diseases of the nervous system and sensory organs (G00-H95)	12 (0.3)	0.68 (0.39-1.20)	13 (0.3)	1.14 (0.66-1.97)
Non-informative causes of death (R00-R94, R95-R99)	63 (1.4)	1.91 (1.49-2.44)	36 (0.9)	2.16 (1.56-3.00)
Unknown [a]	83 (1.8)	-	47 (1.2)	-

[a] Confirmed deaths which could not be assigned to a cause of death

Patients diagnosed 2010-2019 living in North Rhine-Westphalia were included.
Abbreviations: SMR, age-standardised mortality ratio; CI, confidence interval; DCO, death certificate only
